# Comparison of the Effects of Spinal Anaesthesia on Frontal QRS-T Angle in Term and Post-Term Pregnancies Planned for Elective Caesarean Section: A Prospective Study

**DOI:** 10.3390/medicina61050919

**Published:** 2025-05-19

**Authors:** Ahmet Kaya, Mahmut Alp Karahan, Tugba Bingol Tanriverdi, Alev Esercan, Melike Bostanci Erkmen, Zulkif Tanriverdi

**Affiliations:** 1 Department of Anesthesiology and Reanimation, Mehmet Akif Inan Training and Research Hospital, University of Health Science, Sanliurfa 63040, Turkey; mahmutalp_k@yahoo.com (M.A.K.); tuggbabingol@gmail.com (T.B.T.); 2 Department of Obstetrics and Gynecology, Kanuni Training and Research Hospital, University of Health Science, Trabzon 61250, Turkey; alevesercan@gmail.com; 3 Department of Anesthesiology and Reanimation, Sanliurfa Training and Research Hospital, Sanliurfa 63250, Turkey; melikeb63@hotmail.com; 4 Department of Cardiology, Faculty of Medicine, Harran University, Sanliurfa 63300, Turkey; ztverdi@gmail.com

**Keywords:** pregnancy, electrocardiography, QRS duration, frontal QRS-T angle, obstetric anaesthesia, maternal cardiac assessment

## Abstract

*Background and Objectives*: Post-term pregnancies are associated with increased risks of perinatal complications. This study aimed to evaluate potential cardiac electrophysiological changes in pregnant women by comparing the QRS duration, interval of corrected QT (QTc), and frontal QRS-T angle [f(QRS-T)] between term and post-term pregnancies. *Materials and Methods*: In this observational prospective study, 120 pregnant women were enrolled—60 term (37–41 weeks) and 60 post-term (≥42 weeks). All participants underwent standard 12-lead electrocardiography (ECG) and caesarean section with spinal anaesthesia. The QTc interval, QRS duration, and frontal QRS-T angle were measured. Demographic parameters such as age, gestational week, height, and weight were recorded. The SPSS software was used to analyse the data with *p* < 0.05 as the threshold for significance. *Results*: Post-operative QTc interval (417.3 ± 20.5 vs. 410.2 ± 14.5, *p* = 0.032) and f(QRS-T) (28 [16–55] vs. 22 [14–34], *p* = 0.042) were significantly higher in the post-term group than in the term group. When the change in the f(QRS-T) angle was analysed, there was a significant widening of this angle in the post-term group (from 21 [11–37] to 28 [16–55], *p* = 0.002). The increased f(QRS-T) angle reflects greater heterogeneity in ventricular depolarisation and repolarisation, which may indicate sub-clinical myocardial stress or altered autonomic regulation in the post-term period. Although no overt arrhythmias were observed, subtle changes in P-wave morphology and QT dispersion were more prevalent in the post-term group. *Conclusions*: Prolonged QRS duration and an increased f(QRS-T) angle in post-term pregnancies can reflect the underlying changes in cardiac electrophysiology related to prolonged gestation. These ECG parameters may serve as non-invasive indicators of sub-clinical cardiac stress, which could be relevant for anaesthetic risk assessment and perinatal management.

## 1. Introduction

Pregnancy causes numerous alterations in a woman’s physiology. The majority of these alterations are transient, reverting to the pre-pregnancy state within days or weeks following delivery. However, pregnant women who encounter complications during pregnancy may experience symptoms that are unrelated to the normal changes caused by pregnancy. A comprehensive understanding of the haemodynamic changes associated with pregnancy and the peripartum period allows anaesthetists to predict which cardiac lesions may precipitate peripartum haemodynamic deterioration. This knowledge empowers clinicians to guide both anaesthetic management and obstetric emergencies such as emergency caesarean section or post-partum haemorrhage [1].

The cardiovascular system undergoes significant physiological and anatomical changes during pregnancy. There is an increase of 30 to 50% in the amount of blood pumped by the heart (cardiac output). Consequently, the resting heart rate accelerates from a normal pre-pregnancy rate of about 70 beats to up to 90 beats per minute. In addition, during periods of exercise, the cardiac output and heart rate of pregnant women increase to a greater extent than those of non-pregnant women. Concurrently, pregnancy can result in decreases in blood pressure, peripheral resistance, and pulmonary vascular resistance. However, the cardiac conduction system is also affected, which can increase susceptibility to arrhythmias. The most prevalent arrhythmias during pregnancy are atrial arrhythmias; however, while ventricular tachyarrhythmias are rarely observed, they can be life threatening [2].

Caesarean section is one of the most prevalent surgical methods. General anaesthesia, spinal anaesthesia, and epidural anaesthesia can all be utilised in these patients. The use of spinal anaesthesia in caesarean section operations is a frequently employed method due to its swift onset of effect, relative technical ease of application, and higher probability of success. In addition to the aforementioned positive effects, direct cardiac side effects—such as vasodilatation due to sympathetic denervation—and indirect cardiac side effects—such as decreased right heart pressure and reflex bradycardia (Bainbridge reflex)—may be observed in relation to the level of block [3]. It has been demonstrated in previous studies that the administration of spinal anaesthesia is associated with QT prolongation on electrocardiographic (ECG) recordings [4]. Recent evidence has shown that the frontal QRS-T angle [f(QRS-T)] is a more specific marker of myocardial repolarisation than the QT interval. The f(QRS-T) angle is defined as the angular disparity between the direction of ventricular depolarisation (QRS wave) and repolarisation (T wave). The frontal QRS-T angle is a reliable indicator of myocardial electrophysiological instability and is associated with arrhythmias. The f(QRS-T) angle, which is easily obtained from a 12-lead electrocardiogram, is a simple and cost-effective parameter [5].

This study was conducted to investigate how spinal anaesthesia affects the f(QRS-T) angle in term and post-term pregnant women undergoing elective caesarean section.

## 2. Materials and Methods

### 2.1. Ethics

The design of the study was approved by the Clinical Research Ethics Committee at the University of Harran (date: 7 August 2023, number: HRU/23.14.33), and 120 pregnant participants consented. The commencement date for this study is 3 June 2024, and the completion date is 23 December 2024. Our trial is also registered with the Australian–New Zealand Clinical Trials Ethics Committee (Number: ACTRN12624000596505).

### 2.2. Patient Selection

This observational prospective study included American Society of Anesthesiology (ASA) II patients aged 18–45 years undergoing elective caesarean section at Sanliurfa Training and Research Hospital. Patients presenting pre-term pregnancy; multiple pregnancy; foetal anomaly; growth retardation; pathology that may affect the acid–base balance; antepartum haemorrhage; bronchial asthma; Rh incompatibility; peripheral neuropathy; neuromuscular or neuropsychiatric disease; contraindications to spinal anaesthesia; rhythm disturbances and electrolyte disorders; cardiovascular, cerebrovascular, pulmonary, hepatic, and/or renal insufficiency; obesity (body mass index > 30); trauma; cancer; ASA III-IV; and those who did not wish to participate in this study were excluded.

The sample size was calculated based on the results of the first 16 patients who were included in the study. From the observed differences and under the assumption of a two-tailed α-value of 0.05 (sensitivity: 95%) and a β-value of 0.20 (study power: 80%, effect size: 0.52), we determined that at least 120 patients were required for our study (using the G-Power 3 program for power analysis). We decided to enrol at least 60 patients in each group. A total of 160 patients were enrolled in the study, of which 20 patients were excluded because they did not meet the inclusion criteria, 10 patients were excluded because they did not consent, and 10 patients were excluded because the procedure returned to general anaesthesia, leaving 120 patients in the study. Patients were invited to the study according to the order of admission and were included in the groups according to the gestational week at the time of admission. Finally, 60 patients were recruited consecutively for the term group and 60 patients for the post-term group.

### 2.3. Study Design

After obtaining approval from the ethics committee, patients presenting to the Anaesthesia Outpatient Clinic were invited to participate in the study in order of arrival and gestational week. Patients who agreed to participate and met the study criteria were included in the study; they were informed verbally and in writing simultaneously with the anaesthesia consent form, and signed informed consent was obtained.

Those who were at 42 weeks of gestation or greater were defined as group A, while those who were at less than 42 weeks were defined as group B. Post-term pregnant women were referred for caesarean section due to late presentation to hospital, non-progressing labour, and cephalo pelvic disproportion (CPD).

Prior to undergoing surgical procedures, all patients underwent the establishment of vascular access using an 18 G intravenous line with the initiation of a Ringer’s lactate (RL) infusion at a rate of 15 mL/kg/h to facilitate hydration. The patients who were taken to the operating theatre were placed in a decubitus position on the left side and standard monitoring was performed, including non-invasive blood pressure (NIBP), peripheral oxygen saturation (SpO_2_), and electrocardiography (ECG). Baseline systolic (SAP) and diastolic arterial pressure (DAP), mean arterial pressure (MAP), and heart rate (HR) were recorded. Pre-operatively, the initial electrocardiogram (ECG) was obtained in the operating theatre. The patients were seated, and the puncture site was meticulously disinfected with a 10% povidone iodine antiseptic solution. Following the implementation of sterile draping, the subarachnoid space was accessed with a 25 G Quincke needle from the L_3_–L_4_ or L_4_–L_5_ interval. The administration of 12.5 mg of hyperbaric bupivacaine hydrochloride (5 mg/mL, 2.5 mL) was initiated subsequent to the observation of clear cerebrospinal fluid. Subsequent to the application, the patients were positioned in a supine position with a 15° tilt to the left side and a 30° elevation of the head. The attainment of a block height at T_4_, T_6_, or T_10_ dermatome levels was deemed to be adequate for the initiation of surgical intervention.

### 2.4. Measurements

The values of SAP, DAP, MAP, HR, and SpO_2_ were recorded in the immediate period following the administration of the local anaesthetic drug. The time points at which the values were recorded were as follows: the 1st, 5th, 10th, 15th, 20th, 25th, 30th, 35th, 40th, 45th, 50th, and 60th minutes. A decline of 20% in SAP value compared with the initial baseline value, or a decrease in systolic arterial pressure to below 90 mmHg, was indicative of significant hypotension. The protocol stipulated the administration of intravenous fluid infusion and the administration of 10 mg of ephedrine in repeated doses until the blood pressure reached basal values as well as the administration of 0.5 mg atropine when the heart rate was below 50 beats per minute.

### 2.5. Measurement of Electrocardiographic Parameters

ECG (Nihon Kohden, Tokyo, Japan) was performed on two occasions during the operation: once prior to the pre-operative procedure and once at the conclusion of the case. In all patients, a 12-lead ECG was obtained using the following parameters: 25 mm/s paper speed, 10 mm/Mv height, and 0.16–100 Hz filter range. This procedure was conducted in the supine position after a minimum of 10 min of rest.

**Figure 1:**
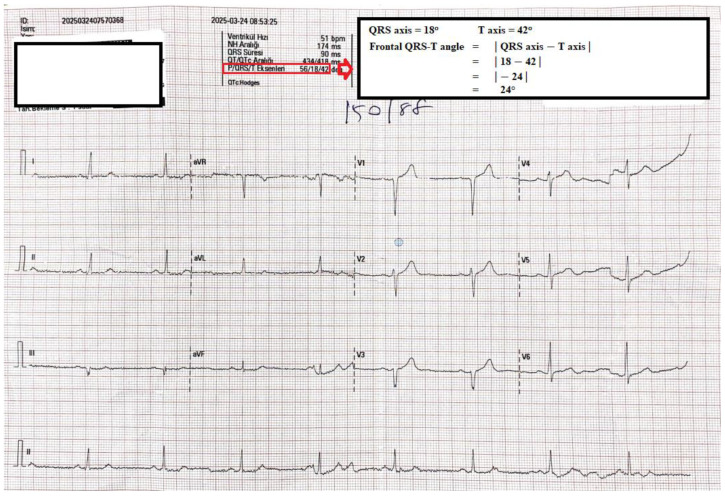
Twelve-lead ECG samples and frontal QRS-T measurement method.

The electrocardiographic evaluation of these patients was performed by two cardiologists. The QT interval’s measurement was conducted with the assistance of magnifiers. The QRS duration, f(QRS-T) angle, QT interval, and corrected QT (QTc) interval parameters were determined from ECGs. The interval between the onset of the Q wave and the termination of the T wave is defined as the QT interval. The calculation of QTc data was performed utilising the Bazett formula [6,7]. The T axis and the fQRS axis were automatically calculated using the reporting section of the ECG device. The cardiologist performed a thorough evaluation of these angles. By calculating the difference between the QRS axis and the T axis, the angle f(QRS-T) is obtained [i.e., f(QRS-T) angle = QRS axis − T axis]. Figure 1 shows the method of measurement of the f(QRS-T) angle that is automatically obtained from the surface ECG device. If this angle is greater than 180°, it is recalculated by subtracting the obtained angle from 360° [8].

### 2.6. Statistical Analysis

SPSS 21.0 was used for statistical analyses. The Kolmogorov–Smirnov test was performed to determine whether the distribution of continuous variables was normally distributed. Continuous variables with normal distribution were evaluated as mean ± standard deviation, and Student’s *t*-test was performed for comparison. Continuous variables without normal distribution are expressed as median (Q1–Q3) and were compared using the Mann–Whitney U-test. Categorical variables are expressed in terms of numbers and percentages. The Chi-square test was used for comparison. For the comparison of two repeated measurements within the same group, if the distribution was normal, a paired sample *t*-test was performed, while a Wilcoxon test was used if the distribution was not normal. A repeated measures of variance test was used to compare two or more repeated measures within the same group. Statistical significance was determined by a *p* value of <0.05.

## 3. Results

The current study included 120 pregnant women undergoing elective caesarean delivery. The baseline clinical characteristics of the post-term and term pregnancies are compared in Table 1, from which it can be seen that there was no significant difference in baseline clinical characteristics between the two groups.

In Table 2, the HR, SpO_2_, and MAP changes during the procedure are shown. There were no significant differences between the groups for MAP, HR, and SpO_2_ at the same time points. MAP significantly decreased in the 1st and 5th minutes; then, it started to increase. HR significantly increased in the 1st minute; then, it decreased gradually with repeated measurements until the post-operative period. However, SpO_2_ values did not significantly change during the procedure.

Electrocardiographic variables at the pre- and post-operative time points are presented in Table 3. Between the two groups, the pre-operative QT values were similar. Pre-operative frontal QRS-T angle and QTc were also similar between the two groups. However, in the post-operative period, the QTc interval (417.3 ± 20.5 vs. 410.2 ± 14.5, *p* = 0.032) and frontal QRS-T angle (28 [16–55] vs. 22 [14–34], *p* = 0.042) were significantly higher in the post-term group compared to the term group. On the other hand, in comparison with the pre-operative values, post-operative QT intervals were significantly increased in both groups, whereas post-operative QTc intervals were not significantly changed in both groups. Analysing the changes in the f(QRS-T) angle, it was significantly widened (from 21 [11–37] to 28 [16–55], *p* = 0.002) in the post-term group, while it did not significantly widen (from 19 [12–23] to 22 [14–34], *p* = 0.117) in the term group.

## 4. Discussion

In this study, we investigated the effect of spinal anaesthesia on the frontal QRS-T angle at term versus post-term in pregnant women undergoing caesarean section. Our main findings were as follows: (I) the f(QRS-T) angle and QTc interval values were significantly higher in the post-term group; and (II) the post-operative QT interval was significantly prolonged in both groups in comparison with pre-operative values.

Arrhythmias are a prevalent complication experienced by anaesthetists during the perioperative period. It is important to consider that factors such as the anaesthetic agents employed, electrolyte values, the insertion of an endotracheal tube and similar events resulting in catecholamine release, along with surgical manipulations, may all contribute to the development of arrhythmia during the perioperative period. One of the most important of these factors is the technique of spinal anaesthesia using bupivacaine. The mechanisms of conduction defects and myocardial depression in arrhythmias related to this technique have been shown to be related to its ability to activate the autonomic nervous system through its effects on beta adrenergic and lysophosphatidate signalling pathways [9].

In order to predict and monitor such arrhythmias, the QT interval—which refers to the time required for the depolarisation of ventricules and repolarisation—can be detected via ECG, which is defined as the time elapsed between the beginning of the QRS complex and the end of the T wave. Discrepancy in the QT interval between derivations signifies a disparity in regional repolarisation. Parameters of ventricular repolarisation (VR), including the QT interval, QT dispersion (QTd), and the value of the QTc interval, have been frequently utilised in studies that seek to estimate the risk of critical ventricular arrhythmias [4,10].

Although cases of caesarean section have been of interest to anaesthetists in terms of investigation of the QT interval, a limited number of studies have been carried out on this subject. In such limited studies, the QT interval during spinal anaesthesia for caesarean section was found to be prolonged in post-term cases with increasing gestational week. Furthermore, the duration of anaesthesia achieved with levobupivacaine compared with bupivacaine was prolonged in measurements taken between the 5th minute after spinal anaesthesia and during skin suturing [4,11]. To the contrary, studies have suggested that epidural anaesthesia for caesarean section has a modulating and reducing effect on the QTc interval in pre-eclamptic patients, and that local anaesthesia has no effect on the QT regardless of the local anaesthetic used [3,12,13].

The frontal QRS-T angle has recently come to fore as an alternative to the QT in terms of resolving this variability in studies [14]. Previous studies have shown that in a variety of cardiovascular diseases, the f(QRS-T) angle has the potential to serve as an independent prognostic marker [15,16,17]. For example, the occurrence of malignant arrhythmias and sudden cardiac death have been associated with a significant increase in the f(QRS-T) angle [8].

Importantly, the spatial QRS-T angle measures electrical activity in the heart and is superior to other conventional ECG parameters. This is because it is a reflection of spatial aspects of the ventricular action potential that are not captured by other parameters [18]. The spatial QRS-T is also less susceptible to measurement error and noise, making it a more robust parameter; in particular, this is due to the location of the peak of T-wave on the ECG. Other ECG measures of the electrical activity of the ventricles—such as the QTc interval and the QT dispersion—are highly dependent on the determination of the wave points of ECG [19]. Other well-known markers of cardiac dysfunction, such as B-type natriuretic peptide and troponin, also differ with respect to the spatial QRS-T angle. Such plasma markers are released by the heart muscle in response to the stretching of and/or damage to the myocardium [20]. However, the spatial QRS-T angle appears to be increased due to structural and/or conduction abnormalities. Therefore, a non-invasive marker for the early detection of subtle cardiac abnormalities is the spatial QRS-T angle [21].

Studies on the effects of anaesthetics or anaesthetic techniques on the f(QRS-T) angle are very rare. Another study examined the effects of body mass index (BMI) on the f(QRS-T) angle in pregnant women who underwent caesarean section with spinal anaesthesia, and they found that the f(QRS-T) angle and QTc interval were significantly increased in pregnant women with BMI ≥ 30 when compared with those with BMI < 30 [22].

Although changes in the QT interval have been demonstrated with an increase in gestational age, studies of changes in the QT interval in cases of caesarean section have yielded inconsistent results. Therefore, our study performed f(QRS-T) measurements to determine the risk of intraoperative arrhythmia in patients due to anatomical and physiological changes predisposing them to proarrhythmic activity with increasing gestational age and maternal haemodynamic changes after spinal anaesthesia. The post-operative QTc interval (417.3 ± 20.5 vs. 410.2 ± 14.5, *p* = 0.032) and f(QRS-T) (28 [16–55] vs. 22 [14–34], *p* = 0.042) were significantly higher in the post-term group than in the term group. On the other hand, the post-operative QT interval was significantly prolonged in both groups in comparison with pre-operative values. When analysing the change in the f(QRS-T) angle, there was a significant widening of the f(QRS-T) angle in the post-term group (from 21 [11–37] to 28 [16–55], *p* = 0.002), whereas no significant widening of this angle was observed in the term group (from 19 [12–23] to 22 [14–34], *p* = 0.117).

For an elective caesarean section, spinal anaesthesia is the most commonly used anaesthetic technique [23], where bupivacaine is the most commonly used local anaesthetic for this technique [24]. It has been reported that both of the physiological changes due to spinal anaesthesia during labour—particularly in the cardiovascular system—and the cardiotoxic effects of bupivacaine used as a local anaesthetic, especially at high dosages, can predispose the patient to arrhythmias. Arrhythmic effects become more pronounced, especially with an increasing dose of the local anaesthetic used. Three doses of bupivacaine have often been used in previous studies on this topic: 15 mg, 12.5 mg, or 10 mg. In studies comparing 15 mg with 10 mg, a prolongation of the QT interval was observed in the 15 mg group [4,13,22]. Although doses of 10 mg or less may lead to conversion to general anaesthesia, we set the dose of bupivacaine that we used in our study at 12.5 mg for both groups.

### Limitations

Our study had a number of limitations: we were only able to obtain two ECG recordings, and f(QRS-T) measured at specific time intervals may not be reliable; in this regard, continuous measurements such as Holter monitoring may be more reliable. A further limitation is that the individual effects of ephedrine, oxytocin, and bupivacaine on the repolarisation of the myocardium were not assessed in the study. Therefore, our results could only be explained according to term vs. post-term pregnancies in women undergoing caesarean section with spinal anaesthesia. If this study had a larger population, it would be possible to compare pregnant women with and without arrhythmias, including those with a high risk of heart disease.

To avoid bias in our study, all pregnant women who were admitted to the hospital on a certain date and met the inclusion criteria were consecutively included in the study groups. Nevertheless, as it was not a randomised trial, the study may have been subject to selection bias. In addition, the QT interval and f(QRS-T) angle may be affected by many confounders and effect-modifying variables, such as age, weight, gender, blood pressure, heart rate, and comorbid diseases. In this study, both groups were similar in terms of these characteristics, but the possibility of residual confounding due to unmeasured covariates cannot be excluded.

## 5. Conclusions

In this study, we showed that spinal anaesthesia widened the post-operative f(QRS-T) angle in caesarean section patients with gestational age ≥42 weeks when compared with the term group. With the results of this study, the decision to perform antenatal delivery in patients with a known history of arrhythmia can be guided by the pre-operative advice of the cardiologist, obstetrician, and anaesthetist. Important cardiovascular changes can be predicted and evaluated before birth and during caesarean section with the aim of preventing maternal mortality due to cardiovascular diseases. Therefore, in patients undergoing caesarean section under spinal anaesthesia—especially those with a gestational age of ≥42 weeks—we recommend post-operative ECG with intraoperative cardiac and f(QRS-T) angle monitoring.

## Figures and Tables

**Table 1:** Comparison of baseline clinical characteristics of the study groups.

Variables	Post-Term Group (n = 60)	Term Group (n = 60)	*p*
Age, years	26.6 ± 4.5	27.3 ± 3.7	0.362 ^a^
Weight, kg	75.9 ± 10.2	73.0 ± 6.0	0.060 ^a^
Height, cm	161.1 ± 5.8	159.5 ± 3.4	0.076 ^a^
Gravida	4 (3–5)	4 (3–6)	0.300 ^b^
Haemoglobin, g/dL	11.2 ± 0.9	11.4 ± 1.3	0.421 ^a^

^a^ Student’s *t*-test, ^b^ Mann–Whitney U-test.

**Table 2:** Mean arterial pressure (MAP), heart rate (HR), and oxygen saturation (SpO_2_) changes in study groups during the procedure.

	Post-Term Group (n = 60)	Term Group (n = 60)	*p*
MAP, mmHG			
Pre-operative	86.3 ± 13.5	87.8 ± 12.8	0.542 ^a^
1st minute	79.9 ± 14.1	81.9 ± 11.2	0.392 ^a^
5th minute	71.6 ± 12.2	75.9 ± 15.5	0.092 ^a^
10th minute	72.3 ± 11.2	75.8 ± 13.7	0.134 ^a^
Post-operative	74.5 ± 11.5	76.8 ± 22.1	0.473 ^a^
	*p* < 0.001 ^c^	*p* < 0.001 ^c^	
HR, /min			
Pre-operative	93.6 ± 8.8	94.7 ± 5.8	0.415 ^a^
1st minute	105.6 ± 15.1	105.9 ± 12.1	0.899 ^a^
5th minute	104.4 ± 17.9	105.6 ± 12.8	0.665 ^a^
10th minute	102.4 ± 16.8	103.9 ± 11.6	0.583 ^a^
Post-operative	88.2 ± 17.9	86.2 ± 24.1	0.600 ^a^
	*p* < 0.001 ^c^	*p* < 0.001 ^c^	
SpO_2_, %			
Pre-operative	98.1 ± 1.0	98.0 ± 1.3	0.575 ^a^
1st minute	98.1 ± 1.1	98.0 ± 1.2	0.387 ^a^
5th minute	98.0 ± 1.2	97.9 ± 1.3	0.614 ^a^
10th minute	97.9 ± 1.1	98.0 ± 1.2	0.937 ^a^
Post-operative	98.3 ± 1.0	97.9 ± 1.3	0.111 ^a^
	*p* = 0.113 ^c^	*p* = 0.181 ^c^	

^a^ Student’s *t*-test, ^c^ repeated measures ANOVA.

**Table 3:** Comparison of QT, corrected QT (QTc), and frontal QRS-T angle changes in the study groups before and after the procedure.

	Post-Term Group (n = 60)	Term Group (n = 60)	*p*
QT, ms			
Pre-operative	345.1 ± 22.4	349.0 ± 20.9	0.326 ^a^
Post-operative	368.1 ± 25.4	364.9 ± 28.2	0.512 ^a^
	*p* < 0.001 ^d^	*p* < 0.001 ^d^	
QTc, ms			
Pre-operative	415.7 ± 22.7	411.9 ± 14.2	0.281 ^a^
Post-operative	417.3 ± 20.5	410.2 ± 14.5	0.032 ^a^
	*p* = 0.751 ^d^	*p* = 0.394 ^d^	
Frontal QRS-T angle (°)			
Pre-operative	21 (11–37)	19 (12–23)	0.349 ^b^
Post-operative	28 (16–55)	22 (14–34)	0.042 ^b^
	*p* = 0.002 ^e^	*p* = 0.117 ^e^	

^a^ Student’s *t*-test, ^b^ Mann–Whitney U-test, ^d^ Paired samples *t*-test, ^e^ Wilcoxon test.

## Data Availability

The original contributions presented in this study are included in the article; further inquiries can be directed to the corresponding author.

## Abbreviations

The following abbreviations are used in this manuscript:

ECG	electrocardiography
f(QRS-T)	frontal QRS-T angle
ASA	American Society of Anesthesiology
RL	Ringer Lactate
SpO_2_	oxygen saturation
NIBP	non-invasive blood pressure
SAP	systolic arterial pressure
DAP	diastolic arterial pressure
MAP	mean arterial pressure
HR	heart rate
QTc	corrected QT
QTd	QT dispersion
BMI	body mass index
